# Sustained shift in the morphology of organic-walled microfossils over the Ediacaran–Cambrian transition

**DOI:** 10.1098/rsos.241966

**Published:** 2025-06-11

**Authors:** Kelly E. Tingle, Ross P. Anderson, Neil P. Kelley, Simon A. F. Darroch

**Affiliations:** ^1^ Department of Earth and Environmental Sciences, Vanderbilt University, Nashville, TN, USA; ^2^ Museum of Natural History, University of Oxford, Oxford, UK; ^3^ All Souls College, University of Oxford, Oxford, UK; ^4^ Paläozoologie, Senckenberg Gesellschaft fur Naturforschung, Frankfurt am Main, Germany

**Keywords:** palaeobiology, micropalaeontology, disparity, morphometrics, Precambrian, Cambrian

## Abstract

The early (approx. 1650–540 Ma) history of eukaryotes was punctuated by several major—but enigmatic—environmental perturbations that potentially influenced the evolution of the Proterozoic biosphere, and the changing structure of Earth systems leading up to the Cambrian Explosion of animals. Reconstructing the manner in which eukaryotes responded to these events represents an innovative lens with which to understand what these perturbations actually represent, as well as the links between geosphere and biosphere during a critical period in eukaryotic evolution. In this study, we analyse organic-walled microfossil size and morphology across the Ediacaran–Cambrian transition. We illustrate that the decrease in vesicle diameter—previously shown to occur across the Ediacaran–Cambrian transition—began in the Ediacaran following the ‘Shuram’ carbon isotope excursion. This size decrease was accompanied by an increase in relative process length across the Ediacaran–Cambrian transition, which has not been previously quantified. Finally, following the ‘Shuram’ excursion, we illustrate a sustained shift in overall morphology. This shift in morphology may have been driven by nutrient stress enhanced by environmental change and/or the increased importance of planktonic lifestyles, highlighting the expansion of microbial eukaryotes into the plankton as a key step in the establishment of modern marine food webs.

## 1. Introduction

**Figure 1.**
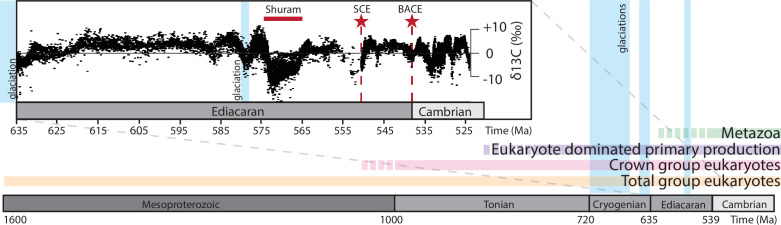
Timeline of early eukaryotic evolution and major Earth system events. Dashed bars represent uncertainty in timing. Early eukaryotes in the form of organic-walled microfossils possess a fossil record [6] that spans critical Earth system events such as global glaciations, the appearance of the first animals and carbon cycle perturbations such as the ‘Shuram’ carbon isotope excursion (red bar), an approx. 550 Ma South China excursion (SCE, red star) and the basal Cambrian carbon isotope excursion (BACE, red star) at the Ediacaran–Cambrian transition. Biomarkers [5] suggest a transition from prokaryote to eukaryote dominated primary production in the early Neoproterozoic. Carbon isotope data from [7].

The Ediacaran–Cambrian transition was a crucial interval in Earth history, witnessing dramatic changes to the structure and function of Earth systems. The aftermath of the Cryogenian Snowball Earth glaciation events was marked by a proliferation in eukaryotic diversity, culminating in the late Ediacaran–Cambrian rise of animals [1–5] (figure 1) and major perturbations to global geochemical cycles. Of particular interest is the Shuram carbon isotope (δ^13^C_carb_) excursion [7–10], which is among the largest negative carbon isotope excursions in Earth history (e.g. [8]), and represents a sustained, globally synchronous perturbation occurring between 574.0 ± 4.7 and 567.3 ± 3.0 millions of years ago (Ma) [9,11]. The links between changes in the biosphere and environmental perturbations (as recorded in isotopic records) are still unclear; however, reconstructing the timing of these events and the response of early eukaryotes is key to understanding the origins of the modern marine biosphere. Here, we compile the record of likely eukaryotic organic-walled microfossils across the Ediacaran–Cambrian transition, and use this dataset to evaluate hypotheses surrounding the influence of environmental perturbations on early eukaryotic evolution.

Organic-walled microfossils of probable eukaryotes are known definitively from sediments as old as approximately 1650 Ma and potentially up to 3200 Ma. They represent our only direct record of early eukaryotic evolution [6,12–14] and have a record spanning the Ediacaran–Cambrian transition and beyond [15–21]. While organic-walled microfossils—which include fossils traditionally termed ‘acritarchs’—are polyphyletic, many have been interpreted as eukaryotic phytoplankton, although some specimens may represent benthic and/or heterotrophic eukaryotes [22–25]. There has been a significant effort to analyse the diversity of this group, both in terms of taxonomic richness, and environmental context across the Proterozoic Eon [6,26–31]. However, the extent to which reconstructed trends in diversity are impacted by biases related to sampling [28,30], taphonomy [28] and taxonomic over-splitting [22,32] remain debated.

An alternative approach to calculating biodiversity as a function of species or generic richness, is quantifying morphological disparity based on morphological character states, which can help avoid biases stemming from taxonomic splitting [32,33]. Analysis of morphological disparity can also reflect changing palaeobiology and ecology; for example, studies of fossil animals and plants suggest that increased disparity can be driven by ecological transitions [34,35], while in protists, disparity has been shown to be influenced by environmental chemistry [36]. Previous work has highlighted the large size and high morphological disparity [33] of early Ediacaran organic-walled microfossils (known as Doushantuo–Pertatataka type acritarchs or ‘DPA’ [37–40])—although some of these may represent the resting stages and embryos of metazoans (i.e. [41,42]). In contrast, late Ediacaran assemblages are usually sphaeromorph dominated, such as the Ediacaran leiosphere palynoflora (ELP), late Ediacaran leiosphere palynoflora (LELP) or terminal Ediacaran *Leiosphaeridia* assemblage (TELA) [37,43]. Cambrian organic-walled microfossils, like those of the early Ediacaran, exhibit high morphological disparity, with a pronounced decrease in vesicle size between the Ediacaran and Cambrian [33], and many early Cambrian organic-walled microfossils have been interpreted as the remains of planktonic microalgae [44–48]. This transition from large taxa in the Proterozoic to small taxa in the Cambrian was suggested by Butterfield [23] to reflect a migration of microbial eukaryotes from the benthos to the plankton.

Given recent discoveries of large, ornamented early Ediacaran ‘DPA’ taxa in late Ediacaran and early Cambrian rocks [16,17,49–51], it is unclear whether previous reconstructions of organic-walled microfossil morphological disparity are accurate. A refined temporal picture of disparity alongside improved high-precision geochronology of the Shuram carbon isotope excursion (Re–Os ages of 574.0 ± 4.7–567.3 ± 3.0 Ma [9]), allows us to re-examine links between environmental perturbations and the diversification of eukaryotes [52–54]. In this study, we reanalyse global morphological disparity among organic-walled microfossils from the Ediacaran to early Cambrian and use these data to evaluate hypothesized links between putative and global pulses of environmental stress and early eukaryotic evolution and ecology.

## 2. Methods

We compiled morphological data from 33 publications on Ediacaran–Cambrian assemblages of organic-walled microfossils (table 1). Although these papers do not encompass every published record of Ediacaran–Cambrian organic-walled microfossils, our analysis required detailed information on fossil size and morphology, meaning that only a subset of the available literature was suitable for this study (i.e. those papers containing detailed systematic descriptions). In addition, some formations, such as the Doushantuo, have been disproportionately studied, leading to an over-abundance of literature based on fossils from this Formation. In an effort to have a more balanced dataset, we therefore limited the contribution of the Doushantuo Formation by only including only a subset of the many publications available (chosen to capture different regions). Even so, Doushantuo occurrences still make up about half of all occurrences within our database, so we applied sensitivity tests (see below) to explore the effects of uneven sampling. Thus, our literature search was constrained, but aimed to capture the full range of taxa and morphologies present within the studied time period. The resulting database includes 635 fossil occurrences from 35 stratigraphic units on five continents.

**Table 1.** Literature from dataset.

Bin	Stratigraphic interval	References	Lithology	Country
before and including Shuram Ediacaran (BSE) 635–567 Ma	Doushantuo Fm.	Xiao *et al*. 2014 [39]	phosphorite	China
		Liu *et al*. 2014 [40]	chert	
		Shang *et al.* 2019 [55]	chert	
		Liu & Moczydłowska 2019 [56]	chert	
	DeyDey Fm.	Willman & Moczydłowska 2011 [57]	siliciclastic	Australia
	Ura Fm.	Sergeev *et a*l. 2011 [58]	siliciclastic	Russia
	Vychegda Fm.	Vorob’eva *et al.* 2009 [38]	siliciclastic	Russia
	Adelaide Rift Complex, Officer Basin, Amadeus Basin	Grey 2005 [37]	siliciclastic	Australia
	Khamaka Fm.	Moczydłowska *et al.* 1993 [59]	siliciclastic	Russia
	Baklia Fm.	Knoll 1992 [60]	chert	Norway
	Dongja Fm.	Yin & Guan 1999 [61]	siliciclastic	China
	Krol A Fm.	Xiao *et al*. 2024 [62]	chert	India
	Infrakrol Fm.	Joshi *et al.* 2016 [63]	chert	India
Post-Shuram Ediacaran (PSE) 566−539 Ma	Stáhpogieddi Fm.	Agić *et al.* 2022 [18]	siliciclastic	Norway
	Dabis Fm.		siliciclastic	Namibia
	Gibbet Hill Fm.		siliciclastic	Canada
	Khesen Fm.	Anderson *et al.* 2019 [49]	phosphorite	Mongolia
	Bocaina Fm.	Morais *et al.* 2021 [51]	phosphorite	Brazil
	Frecheirinha Fm.	Chiglino *et al.* 2015 [64]	carbonate	Brazil
	Matjies River Fm. Groenefontein Fm. Huis River Fm.	Gaucher & Germs 2006 [65]	carbonate	South Africa
			siliciclastic	
			siliciclastic	
	Mortensnes Fm.	Agić *et al.* 2024 [21]	siliciclastic	Norway
	Sete Lagoas Fm.	Denezine *et al*. 2024 [66]	carbonate	Brazil
	Dracoisen Fm.	Knoll & Swett 1987 [67]	siliciclastic	Norway
	Lublin Fm.	Moczydłowska 1991 [68]	siliciclastic	Poland
Early Cambrian (EC) 538−509 Ma	Lukati Fm.	Moczydłowska 2011 [47] Agić 2016 [44]	siliciclastic	Estonia
	Buen Fm.	Wallet *et al.* 2023 [20]	siliciclastic	Greenland
	Yurtus Fm.	Yao *et al*. 2005 [69]	chert	China
	Xishanblaq Fm.		chert	China
	Tokammane Fm.	Knoll & Swett 1987 [67]	siliciclastic	Norway
	Mazowsze Fm. Kaplonosy Fm. Radzyń Fm.	Moczydłowska 1991 [68]	siliciclastic	Poland
	Ella Island Fm.	Vidal 1979 [70]	siliciclastic	Greenland
	Yanjiahe Fm.	Ahn & Zhu 2017 [71] Shang *et al.* 2020 [72]	chert	China
	Grammajukku Fm.	Moczydłowska 2002 [73]	siliciclastic	Sweden
	Hanford Brook Fm.	Palacios *et al.* 2016 [74]	siliciclastic	Canada
	Balang Fm.	Yin *et al.* 2021 [75]	siliciclastic	China

We scored taxa for two continuous characters—vesicle diameter and the ratio of process length to vesicle diameter (processes or spines are protrusions from the cell wall—organic-walled microfossils with processes are called ‘acanthomorphic’)—and 23 presence/absence characters largely focussed on process morphology (mostly taken from [33]; electronic supplementary material, figure S1; table 2) based on published descriptions. If vesicle diameter and process length were reported as mean values in the original publication, these were used. However, if these data were reported as a range of minimum and maximum values, we calculated the midpoint. The ratio of process length to vesicle diameter was calculated using either the reported means, or calculated midpoint in the range of each parameter.

**Table 2.** Morphological characters used in analysis. See supplementary material, figure S1 for illustration of morphological characters.

Character	Coding
vesicle size	1 = <100 µm, 2 = 100–200 µm, 3 = >200 µm
process length	0 = absent, 1 = <10%, 2 = 10–40%, 3 = >40%
irregularly distributed processes	0 = absent, 1 = present
densely distributed processes	0 = absent, 1 = present
branching processes	0 = absent, 1 = present
hollow processes	0 = absent, 1 = present
cylindrical processes	0 = absent, 1 = present
bulbous processes	0 = absent, 1 = present
hooked processes	0 = absent, 1 = present
conical processes	0 = absent, 1 = present
domed processes	0 = absent, 1 = present
heteromorphic processes	0 = absent, 1 = present
processes with expanded base	0 = absent, 1 = present
thin processes (<1 µm)	0 = absent, 1 = present
processes with blunt tips	0 = absent, 1 = present
processes with pointed tips	0 = absent, 1 = present
processes with rounded tips	0 = absent, 1 = present
processes with flared tips	0 = absent, 1 = present
processes communicate with vesicle interior	0 = absent, 1 = present
processes within outer wall	0 = absent, 1 = present
flange	0 = absent, 1 = present
surface ornamentation	0 = absent, 1 = present
colonial or aggregate	0 = absent, 1 = present

Microfossil occurrences were split into three chronostratigraphic bins, based on the most rigorous age estimates for their host geological units taken from recent literature. The bins subdivided the Ediacaran period into BSE (before and including the ‘Shuram’ excursion [635−567 Ma]) and PSE (Post the ‘Shuram’ excursion [566−539 Ma]), with the third-time bin EC (early Cambrian [538–509]).

Mean and median vesicle diameter, process length, and the ratio between process length and vesicle diameter were calculated across all occurrences for each geochronological bin with the data presented as box plots with first and third quartiles. We used nonmetric multidimensional scaling (NMDS) to create a two-dimensional ordination from the 23-character matrix of 635 occurrences, with 20 iterations performed (stress = 0.17 [a measure of induced distortion; though see [76]], *r*^2^ = 0.97, electronic supplementary material, figure S2). We used permutational multivariate analysis of variance to calculate a Bray−Curtis dissimilarity matrix and compare the centroids of our time bins in morphometric space. Spearman’s coefficients were used to determine covariance between the 23 variables used in the morphometric analysis. To estimate disparity within time bins, we used a multisite index analogue of beta diversity (Sorenson’s), where taxa are treated as localities, and characters are treated as species. All analyses were performed using the statistical computing software R, v. 4.3.1. [77].

### 2.1. Sensitivity analyses

We performed a suite of sensitivity analyses, outlined in the following sections, to estimate the impact of various forms of bias on our results.

#### 2.1.1. Impact of sampling bias—removal of Doushantuo Formation and rare occurrences

Estimates of taxonomic diversity—and thus potentially also morphological disparity—can be heavily influenced by a number of geological and sampling-related biases. These include the number of formations in time bins, variable preservation among formations (i.e. ‘lagerstätte effects’), and the extent to which these formations have been more or less intensively sampled (i.e. ‘worker effort’, see [78–82]). Several such problems are potentially present in our data; for example, each of our time bins are characterized by different numbers of formations. In addition, the Doushantuo Formation (BSE) is extremely well-sampled (accounting for 51% of our dataset), and thus might be expected to exert an overwhelming influence on overall patterns. Moreover, some formations preserve either some species in extremely low abundances, or only one described species—suggesting variable preservation of rare species and/or variable sampling among formations. To test whether any observed trends in morphological disparity are driven by differences in sampling among time bins and formations, and/or the overwhelming influence of a small number of intensively-studied formations, we perform two sensitivity tests, by first excluding data from the Doushantuo Formation and second excluding taxon occurrences with <10 specimens reported.

#### 2.1.2. Impact of sampling bias—iterative subsampling

We performed four subsampling routines on our dataset to explore the impacts of raw alpha diversity differences, rare species and poorly sampled formations on overall patterns in size or proportions (e.g. [82]). First, we iteratively (1000×) subsampled 5, 8 and 10 species from each time bin to control for the influence of ‘rare’ species. Second, following the same procedure, we subsampled after removing Doushantuo Formation data. Third, we then subsampled again (excluding Doushantuo) and removed units that preserve only one species. Last, we subsampled after re-including Doushantuo but excluding units that preserve only one species.

#### 2.1.3. Impact of lithological bias

Secular changes in taphonomic and/or palaeoenvironment context may impact these analyses, as different lithologies can preserve organic-walled microfossils with varying fidelity. To better understand the influence of lithological bias on size, we binned occurrences into four lithological categories—siliciclastics, carbonates, cherts and phosphorites—and examined size over the studied interval within individual rock types.

#### 2.1.4. Impact of temporal binning

To assess the sensitivity of our choice of time bins on the patterns observed, we also examined size through time by formation. We calculated the median age of each formation (see electronic supplementary material) after compiling available age constraints and plotted mean vesicle diameter and the ratio of process length to vesicle diameter for each formation (electronic supplementary material, figure S8). Similarly, we then analysed size, and morphological disparity was analysd with only two time bins—Ediacaran (635–539 Ma) and early Cambrian (538–509).

#### 2.1.5. Impact of size on morphometric analyses

Morphological disparity was analysed using a modified character matrix of the entire dataset without the two continuous size characters to determine the impact of organism size in driving overall morphological disparity in these analyses.

## 3. Results

**Figure 2.**
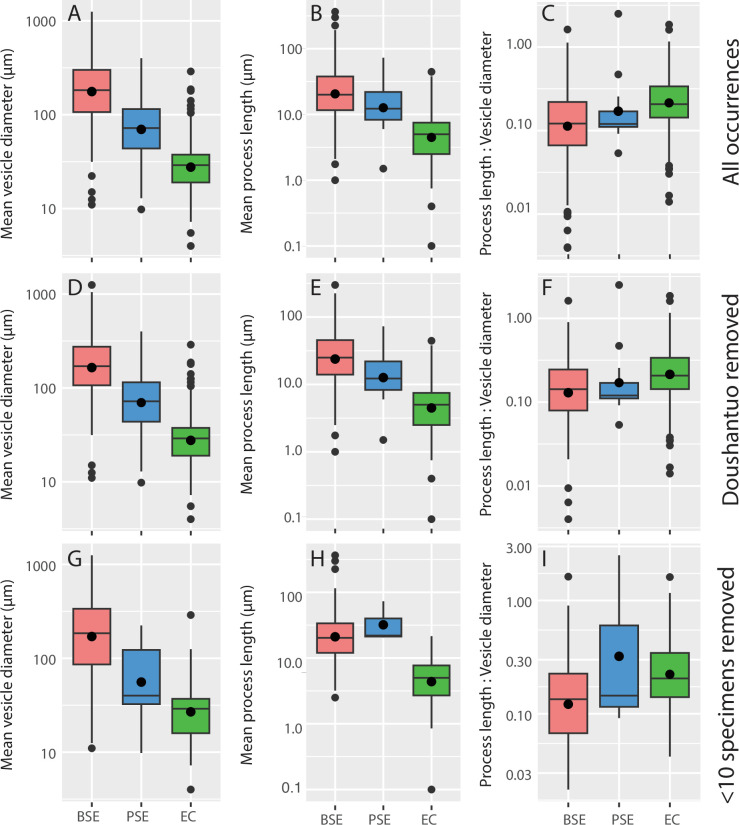
Size and proportion history of organic-walled microfossils across the Ediacaran–Cambrian transition. Upper and lower bounds are the first and third quartiles (the 25th and 75th percentiles), and lines and dots within box plots are median and mean values, respectively. (A) Mean vesicle diameter decreases stepwise in organic-walled microfossils from before the ‘Shuram’ carbon isotope excursion in the Ediacaran (BSE, pink), to post-Shuram Ediacaran (PSE, blue), to early Cambrian (EC, green) when analysing the full dataset. (B) Mean process length decreases across time bins. (C) Proportional process length (the ratio of process length to vesicle diameter) increases from BSE to EC time bins. (D-F) Trends in mean vesicle diameter, process length and proportional process length across time bins with all data except Doushantuo occurrences are similar to trends observed in the raw data. (G–I) Mean vesicle diameter, process length and proportional process length across time bins excluding rare species (species in dataset with less than 10 observed specimens) show similar patterns in BSE and EC organic-walled microfossils, although there is more variance in PSE organic-walled microfossils in this subsampled analysis.

Mean vesicle diameter decreases markedly throughout the studied interval, from 230 µm BSE, to 96.4 µm PSE, and finally to 35.6 µm EC (figure 2A) and pairwise comparisons between all-time bins are statistically significant (table 3). Mean process length also decreases from 33.2 µm BSE, to 18.8 µm PSE, and finally to 6.26 µm EC (figure 2B; table 3). However, the ratio of process length to vesicle size increases across time periods and is statistically significant between BSE and EC time bins (figure 2C; table 3).

**Table 3.** A full report of all size analyses including mean values, s.d., sample size and statistically significant Mann–Whitney test *p*-values (i.e. no value is reported when not significant).

Sample size	Time bin	Measurement	Mean	*n*	s.d.	*p*‐value
all data	BSE	vesicle diameter	230 µm	353	179 µm	BSE–PSE = 1.4 × 10^−11^
						BSE–EC = 2.2 × 10^−16^
	PSE		96.4 µm	48	85.3	PSE–EC = 1.6 × 10^−12^
	EC		35.6 µm	233	32.0 µm	
	BSE	process length	33.2 µm	300	41.6 µm	BSE–EC = 2.2 × 10^−16^
						PSE–EC = 3.1 × 10^−4^
	PSE		18.8 µm	12	19.0 µm	
	EC		6.26 µm	153	6.07 µm	
	BSE	process length: vesicle diameter	0.17	304	0.18	BSE–EC = 2.0 × 10^−12^
	PSE		0.35	12	0.68	
	EC		0.29	152	0.27	
Doushantuo excluded	BSE	vesicle diameter	221 µm	148	189 µm	BSE–PSE = 3.3 × 10^−9^
						BSE–EC = 2.2 × 10^−16^
	BSE	process length	39.2 µm	113	46.7 µm	
	BSE	process length: vesicle diameter	0.19	116	0.20	BSE–EC = 1.3 × 10^−5^
rare (<10) organic-walled microfossils excluded	BSE	vesicle diameter	248 µm	121	222 µm	BSE–EC = 2.2 × 10^−16^
						PSE–EC = 0.006
	PSE		82.8 µm	11	75.3 µm	
	EC		34.8 µm	130	32.4 µm	
	BSE	process length	35.6 µm	94	54.2 µm	
	PSE		38.4 µm	3	29.8 µm	
	EC		5.70 µm	83	4.06 µm	
	BSE	process length: vesicle diameter	0.18	94	0.21	BSE–EC = 3.5 × 10^−6^
	PSE		0.91	3	1.38	
	EC		0.28	82	0.26	
subsampling routine #1	BSE	process length: vesicle diameter	0.17 (5 sp.)	1000	0.08	BSE–PSE = 2.2 × 10^−16^ (5 sp.)
			0.17 (8 sp.)	1000	0.06	BSE–EC = 2.2 × 10^−16^ (5 sp.)
			0.18 (10 sp.)	1000	0.06	
	PSE		0.35 (5 sp.)	1000	0.23	PSE–EC = 4.7 × 10^−5^ (5 sp.)
			0.35 (8 sp.)	1000	0.14	
			0.36 (10 sp.)	1000	0.09	
	EC	0.29 (5 sp.)	1000	0.11		
		0.29 (8 sp.)	1000	0.09		
		0.29 (10 sp.)	1000	0.09		
subsampling routine #2	BSE	process length: vesicle diameter	0.20 (5 sp.)	1000	0.09	BSE–PSE = 2.2 × 10^−16^ (5 sp.)	
			0.19 (8 sp.)	1000	0.07		
			0.20 (10 sp.)	1000	0.07	BSE–EC = 2.2 × 10^−16^ (5 sp.)	
	PSE		0.34 (5 sp.)	1000	0.23	PSE–EC = 9.5 × 10^−6^ (5 sp.)	
			0.36 (8 sp.)	1000	0.14		
			0.36 (10 sp.)	1000	0.09		
	EC		0.29 (5 sp.)	1000	0.12		
			0.29 (8 sp.)	1000	0.09		
			0.29 (10 sp.)	1000	0.08		
subsampling routine #3	BSE	process length: vesicle diameter	0.18 (5 sp.)	1000	0.10	BSE–PSE = 2.2 × 10^−16^ (5 sp.)	
			0.19 (8 sp.)	1000	0.08	BSE–EC = 2.2 × 10^−16^ (5 sp.)	
	PSE		0.17 (5 sp.)	1000	0.04		
			0.17 (8 sp.)	1000	0.01		
	EC		0.28 (5 sp.)	1000	0.11		
			0.28 (8 sp.)	1000	0.08		
subsampling routine #4	BSE	process length: vesicle diameter	0.17 (5 sp.)	1000	0.09	BSE–EC = 2.2 × 10^−16^ (5 sp.)	
			0.17 (8 sp.)	1000	0.07		
	PSE		0.17 (5 sp.)	1000	0.04	PSE–EC = 2.2 × 10^−16^ (5 sp.)	
			0.17 (8 sp.)	1000	0.02		
	EC		0.29 (5 sp.)	1000	0.12		
			0.29 (8 sp.)	1000	0.09		
phosphorites	BSE	vesicle diameter	298 µm	42	203 µm		
	PSE		149 µm	18	108 µm		
	BSE	process length	20.7 µm	32	14.3 µm		
	PSE		16.1 µm	9	8.19 µm		
	BSE	process length: vesicle diameter	0.11	32	0.06		
	PSE		0.16	9	0.12		
cherts	BSE	vesicle diameter	237 µm	189	177 µm	BSE–EC = 4.4 × 10^−6^	
	EC		37.7 µm	10	88.3 µm		
	BSE	process length	31.1 µm	154	41.2 µm		
	EC		5.88 µm	5	4.52 µm		
	BSE	process length: vesicle diameter	0.17	154	0.18	BSE–EC = 0.005	
	EC		0.64	5	0.50		
siliciclastics	BSE	vesicle diameter	196 µm	122	165 µm	BSE–PSE = 4.6 × 10^−7^	
						BSE–EC = 2.2 × 10^−16^	
	PSE		62.0 µm	34	49.8 µm	PSE–EC = 0.001	
	EC		35.5 µm	223	27.5 µm		
	BSE	process length	42.4 µm	91	50.3 µm		
	PSE		6.5 µm	1	—		
	EC		6.30 µm	144	6.14 µm		
	BSE	process length: vesicle diameter	0.22	94	0.21		
	PSE		.11	1	—		
	EC		0.27	143	0.23		
carbonates	PSE	vesicle diameter	69.0 µm	12	44.1		
	PSE	process length	37.1 µm	2	50.4 µm		
	PSE	process length: vesicle diameter	1.30	2	1.69		
Ediacaran–Cambrian time bins only	ediacaran	vesicle diameter	214 µm	401	178 µm	E–C = 2.2 × 10^−16^	
		process length	32.6 µm	312	41.1 µm	E–C = 2.2 × 10^−16^	
		process length: vesicle diameter	0.18	316	0.22	E–C = 1.9 × 10^−12^	

**Figure 3.**
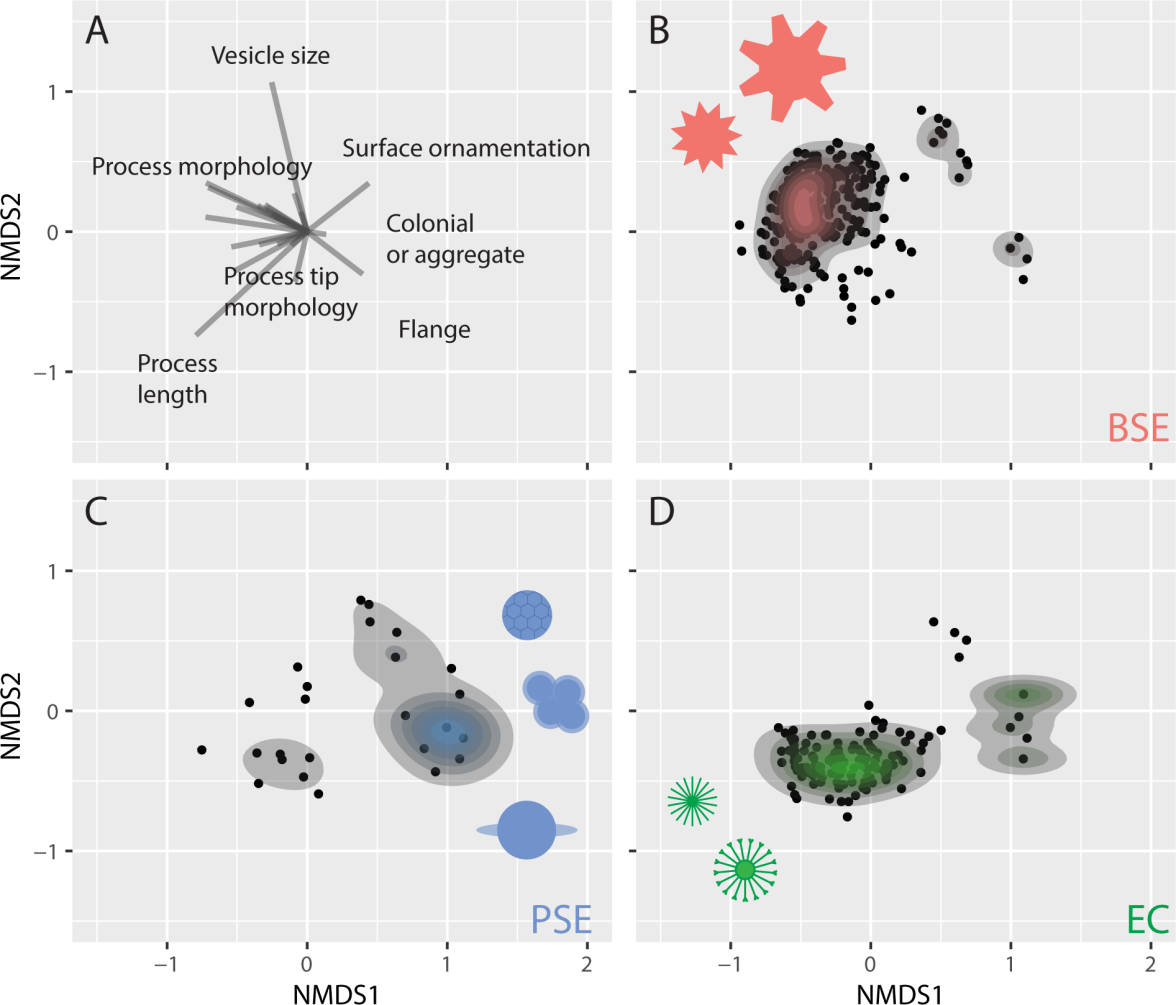
Total occupied morphological space visualized with nonmetric multidimensional scaling of 23 characters from 635 taxa. Each time bin illustrates distinct areas of occupied morphological space. Saturation of colour indicates higher density of points in the contour plots. Cartoons illustrate example morphologies. (A) Illustration of multivariable loadings. (B) BSE taxa cluster in the upper left quadrant, reflecting a diversity of process morphologies and large vesicle size. (C) PSE taxa occupy a transitional area between BSE and EC taxa and are influenced by surface ornamentation, colonial/aggregate occurrences, flange (an equatorial membrane, See electronic supplementary material, figure S1) morphotypes and longer relative process length. (D) EC occurrences cluster in the middle bottom left quadrant due to smaller vesicle size, increased process length and diverse process tip morphologies, with moderate contribution from flange and colonial/aggregate morphotypes.

Organic-walled microfossils from each of the three intervals span a wide range of values in morphological space across two NMDS axes, however, BSE and EC time bins occupy largely distinct areas, with PSE occupying a transitional area between the two (figure 3). These results parallel differences in vesicle diameter and process length reported above, and permutational multivariate analysis of variance indicates that the differences between time bin centroids are statistically significant (*p*‐value = 0.001). Morphospace among BSE taxa (figure 3B) is driven by increased vesicle size and diverse process morphologies (see electronic supplementary material, figure S1). EC morphospace (figure 3D) is driven by increased process length, a diversity of process tip morphologies and an increased importance of flange (an equatorial membrane surrounding vesicles, see electronic supplementary material, figure S1) and colonial/aggregate morphotypes and occurrences (see electronic supplementary material, figure S1). PSE morphospace (figure 3C) falls between the two end members of BSE and EC, occupying a transitional area driven by flange and colonial/aggregate morphotypes and occurrences, increased process length, and surface ornamentation. Within-time bin disparity (quantified here using an index of beta diversity) is highest in BSE, decreases in PSE, and increases—although not to BSE levels—in EC (electronic supplementary material, figure S3).

A correlation matrix of all characters used in this study (electronic supplementary material, figure S4) shows that some characters were highly correlated with each other; e.g. processes that communicate with the vesicle interior tend to be hollow, conical and pointed. However, other characters were negatively correlated; surface ornamentation, for example, is rarely observed on organic-walled microfossils with hollow processes.

### 3.1. Impact of sampling bias—removal of Doushantuo Formation and rare occurrences

Trends in vesicle size and process length are consistent with patterns observed in the entire dataset when occurrences from the Doushantuo Formation are removed (figure 2D–F; table 3). When occurrences with <10 specimens are removed, vesicle diameter also shows broadly similar trends to the full dataset (figure 2G), although only the decrease in vesicle diameter from BSE to the EC is significant (table 3), and trends in proportional process length are similar to patterns observed in the full dataset (table 3; figure 2H–I). In morphometric analyses, the removal of occurrences from the Doushantuo Formation (electronic supplementary material, figure S5A–S5D) produces similar patterns of morphospace occupation between time bins as the entire dataset apart from an axis rotation typical for NMDS. Excluding occurrences with <10 reported specimens also yields broadly similar results although the sample size for PSE is substantially reduced leading to a contraction in total morphospace for this interval (electronic supplementary material, figure S5E–S5H).

### 3.2. Impact of sampling bias—iterative subsampling

Subsampling routine #1 (1000× selecting 5, 8 and 10 species from each time bin) illustrates an increase in the ratio of process length to vesicle diameter with time, however, this increase occurs across BSE (0.17, 0.18)–PSE (0.35, 0.36) time bins and is sustained in EC (0.29), which is in contrast to the stepwise increase observed in the raw data (electronic supplementary material, figure S6A–S6C). Subsampling routine #2 (iterative subsampling with the removal of Doushantuo occurrences) shows a similar pattern, although mean BSE relative process length increases only slightly to 0.20 (electronic supplementary material, figure S6D–S6F). This trend changes in subsampling routine #3 (removing Doushantuo Formation and formations with only one species), where relative process length decreases from BSE (0.18, 0.19) to PSE (0.17) and then increases in EC (0.28) (electronic supplementary material, figure S6G–S6H). Finally, in subsampling routine #4 (including data from the Doushantuo Formation, but excluding formations with only one species), relative process length is the same in BSE and PSE time bins (0.17) and increases in the EC (0.29) (electronic supplementary material, figure S6I, J), thus producing similar results as routine #3.

When comparing these size sensitivity tests, it is apparent that variance in our PSE time bin is heavily influenced by three formations possessing only one acanthomorphic taxon each, which have an outsized effect on perceived trends in the ratio of process length to vesicle size. Subsampling routines which exclude formations with only one acanthomorphic species show a delayed increase in relative process length appearing after the Ediacaran–Cambrian boundary (e.g. compare electronic supplementary material, figure S6A versus electronic supplementary material, figure S6G). At least two of these singleton taxa from the PSE could also be excluded due to poor age constraints (*Asteridium* cf. *A. tornatum*, Matjies River Formation [65]) and possible reworking (‘Unnamed Form E’, Mortenses Formation [21]). When these factors are considered, what we consider to be the most conservative and robust result revealed in the data is close to our ‘raw’ pattern but involves a relatively uncertain or small increase in process length–vesicle diameter ratio over the BSE–PSE transition, and a more certain and much larger increase over the entire BSE through EC transition.

### 3.3. Impact of lithological bias

Restricting occurrences within single lithologies (electronic supplementary material, figure S7) largely preserves the same trends in morphology as shown in the raw data (figure 2). Only siliciclastic occurrences appear in all three bins and carbonates are only found in the PSE bin. Chert comprises the largest fraction of BSE occurrences but is absent from PSE and forms a small fraction of EC occurrences, whereas phosphorites occur as subordinate fractions in BSE and PSE, but not EC, bins (electronic supplementary material, figure S7A). Individual taxa display similar mean vesicle diameters when compared among differing lithologies (electronic supplementary material, figure S7B–S7E) and within time bins, and trends in these mean vesicle diameters between time bins mirror those for the entire dataset (table 3). The ratio of process length to vesicle diameter both within and between time bins is also similar across organic-walled microfossils from different lithologies (electronic supplementary material, figure S7J–S7M), although is longer in cherts (0.17 BSE, 0.64 EC) compared to siliciclastics (0.22 BSE, 0.27 EC); thus, it is possible that long, thin processes may only be preserved through authigenic mineralization, i.e. replacement by silica or phosphate minerals [83,84].

### 3.4. Impact of temporal binning

Rerunning our analyses at the resolution of geological formations (electronic supplementary material, figure S8) lends further support to the inference that vesicle size decreases following the Shuram excursion, followed by a second decrease after the Ediacaran–Cambrian boundary. We concede, however, that some formations—in particular the Vychegda, Matjies River, Groenefontein, Huis Riviere, Frecheirinha formations—have relatively poor radiometric age constraints (potentially straddling boundaries between BSE, PSE and EC), and thus the temporal relationship between size change and the Shuram excursion is sensitive to these specific dates. Taxa within the Vychegda formation are shared with BSE formations elsewhere such as China and Australia, supporting a BSE interpretation for this formation and consistent with the high vesicle diameters observed within this unit [38]. Similarly, analysing ratios of process length to vesicle size data in this fashion lends support to the inference of increasing relative process length across the Ediacaran–Cambrian boundary. Although much of this signal is driven by the Xishanblaq and Yurtus formations (approx. 530 Ma), we note that taxa from the later Cambrian also tend to possess longer relative process lengths that PSE or BSE taxa (electronic supplementary material, figure S8).

When using only Ediacaran and early Cambrian time bins, pairwise comparisons between Ediacaran mean vesicle diameter (214 µm), process length (33 µm) and relative process length (0.18), and those of early Cambrian organic-walled microfossils were statistically significant (electronic supplementary material, figure S9A–S9C; table 3). NMDS morphological disparity analyses show a shift in occupied morphological space between Ediacaran and early Cambrian time bins—largely influenced by increased process length and a decrease in process diversity—although there is moderate overlap between the two bins (electronic supplementary material, figure S9D–S9F).

### 3.5. Impact of size on morphometric analyses

When we excluded continuous size characters from our disparity analyses (electronic supplementary material, figure S5I–S5J), BSE and EC are mostly disparate in morphological space, with PSE occupying a transitional area; BSE morphospace is influenced by a diversity of process and process tip morphologies, while EC morphospace is more influenced by funnel-tipped processes, flange and colonial/aggregate morphotypes and occurrences.

## 4. Discussion

**Figure 4.**
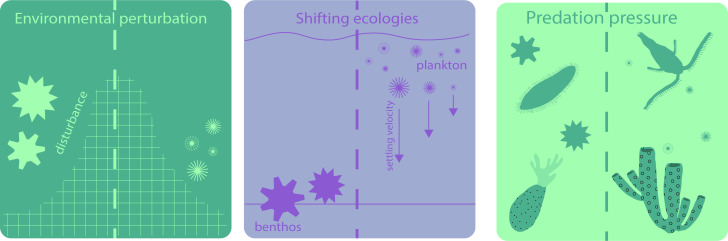
Possible external influences on morphology of organic-walled microfossils over the Ediacaran–Cambrian transition. Environmental perturbations over the Ediacaran–Cambrian transition such as perturbations to the carbon cycle (‘Shuram’ carbon isotope excursion), anoxic events and other essential nutrient limitation, would have placed selective pressure on larger-sized organisms. Expansion into new ecological niches, such as a transition from benthic to planktonic life modes, could explain a transition to smaller vesicle size with proportionally longer processes, as these morphologies would promote suspension in the water column. Escalating predation pressure may have also influenced morphology through the adaptation of features that would have increased sinking rates and therefore evaded grazing predators.

Our analysis illustrates a sustained change in organic-walled microfossil morphology from large morphotypes with diverse but short spine morphologies in the early Ediacaran, to small morphotypes with proportionately long, thin spines in the early Cambrian. Although these changes in disparity are broadly consistent with previous studies—corresponding to a perceived drop in species richness across the Ediacaran–Cambrian boundary [6,26,27,33] (although more recently shown to have occurred after the Gaskiers glaciation [31])—our analysis newly illustrates that this morphological shift begins in the Ediacaran in the aftermath of the Shuram excursion, with a sustained shift across the Ediacaran–Cambrian transition (figures 2 and 3). The observed trends in vesicle size and morphological disparity are robust to sensitivity tests, remaining little-changed when we account for several potential sources of bias. This pattern is consistent with a scenario in which the environmental perturbations that characterized the Ediacaran–Cambrian transition had global scale effects on the morphological character of organic-walled microfossils and thus—potentially— eukaryote ecology, with wide-ranging implications for planktonic food webs and the structure of global biogeochemical cycles (figure 4).

The time interval covered in our dataset is punctuated by at least three intervals of global environmental perturbation—the Shuram excursion, the late Ediacaran (and informally named) ‘White Sea-Nama’ transition, and the Ediacaran–Cambrian boundary. Each of these may have contributed to biotic turnover and morphological change—similar to what has been reported for acritarchs across the end-Ordovician [85] and Permian-Triassic [86] events. We concede that the choice of time binning intervals could have created an artificial stepwise pattern in the data; however, when viewing size by formation, patterns are broadly consistent with our binned analyses (electronic supplementary material, figure S8), and splitting Ediacaran occurrences into pre- and post-Shuram intervals reveals a nuanced morphological shift that is obscured by lumping Ediacaran occurrences into one time bin (compare figure 3B–D versus electronic supplementary material, figure S9E,F).

The cause of the Shuram carbon isotope excursion remains contentious and hypotheses on its origins point to changes in marine redox conditions (e.g. [31,52,87,88]), primary productivity [10], eustatic sea level [10], nutrient availability [89] or—alternatively—that it represents a diagenetic event (e.g. [90,91]). Evidence for environmental change over the White Sea-Nama transition at approximately 548 Ma is similarly enigmatic. This interval corresponds to a decrease in the diversity of soft-bodied macrofossils (the ‘Ediacara biota’), coincident with the rise of the latest Ediacaran ‘Wormworld’ metazoan fauna and appearance of benthic communities that closely resemble the basal Cambrian [3,92,93]. Although some studies [92,94,95] have attributed this turnover to a rise in metazoan ecosystem engineering, Yang *et al.* [53] found evidence for a negative carbon isotope excursion in South China that ended at approximately 550 Ma. This excursion is potentially correlative with excursions found in Namibia and the western United States [53] and may represent a pulse of marine anoxia [96,97]. Lastly, the E–C boundary at approximately 539 Ma (although potentially younger than 533 Ma; see [98]) is more definitively linked with environmental perturbations, as suggested by the basal Cambrian carbon isotope excursion, a large and near-global negative carbon isotope excursion with δ^13^C_carb_ values <6‰; see [99]. Coeval with the E–C boundary, there is growing evidence for rift-related volcanism in Laurentia [100], and an apparent pulse of extinction that removed both shelly fauna and the last of the Ediacara biota [92,101,102].

The late Ediacaran and early Cambrian environmental perturbations listed above may have caused a variety of ecological stresses that could be causally linked to the observed shifts in microfossil disparity. For example, geochemical evidence suggests that essential nutrients like nitrogen, during the Shuram excursion [89], and oxygen, during the Ediacaran–Cambrian boundary [97,103,104] were limited, and Agić *et al.* [21] hypothesize that organic-walled microfossil turnover observed following the Shuram excursion (or possibly even contemporaneous with the excursion) may be linked to nutrient depletion associated with glaciations [105]. Limitation of essential nutrients may have led to selective pressure on larger-sized microbial organisms, as many essential physiological functions of plankton such as metabolic rate, nutrient diffusion and light adsorption are directly related to size [106]. In addition, modern plankton can use spines to increase cellular surface area in contact with the environment to better perceive chemical and physical changes [107] and spines can modify tumbling and therefore diffusion rates [108]. We suggest that the data presented here is consistent with nutrient limitation, as a decrease in vesicle diameter and increase in relative process length would increase the amount of surface area per volume available for gas exchange and nutrient diffusion.

Consequently, we argue that these findings lend support to interpretation of the Shuram excursion as recording environmental perturbation and linked with a biotic turnover event. We also find evidence of continued morphological change among organic-walled microfossils into the Cambrian; our choice of time bins may induce the stepwise pattern shown in our results, although this pattern is consistent when we reanalyse data at the level of geological formations (electronic supplementary material, figure S7). Putative cause-and-effect links between enigmatic Neoproterozoic environmental perturbations and pulses of biotic turnover that have been suggested in the past [21,26,96] are thus supported by focused analysis.

Regardless of driver, the resulting shifts in disparity suggest implications for both the changing character of food webs, and in turn the structure of global biogeochemical cycles. Butterfield [23] hypothesized that the observed transition from large taxa in the Proterozoic to small taxa in the Cambrian reflects an ecological shift to an increasing importance of planktonic (versus benthic) life modes, and thus an increasing role of eukaryotes in global primary production—something also reflected in biomarker data [5,109]. In fact, many early Cambrian organic-walled microfossils have been interpreted as the remains of planktonic green microalgae [44–48]. Modern phytoplankton cell size ranges from picometers up to 200 µm (e.g. [106]). PSE and EC organic-walled microfossils are well within these limits, averaging 96 and 36 µm, respectively (figure 2; table 3). BSE microfossils, by contrast, have a mean vesicle size (230 µm) outside of the typical size range for modern phytoplankton. Cohen *et al.* [41] hypothesized that early Ediacaran acanthomorphs such as *Appendisphaera, Tanarium* and *Tianzshuania* represent the remains of resting stages of animals based on a comparable size range in addition to their morphology, and wall ultrastructure. Given our data, it is possible that some taxa within the BSE population (figure 3B) represent an ecologically or phylogenetically distinct, benthic and/or possibly eumetazoan population separate from PSE and EC taxa, which may predominantly represent planktonic protists.

The smaller organic-walled microfossils present in this study—if part of the planktonic community—may have evolved process morphologies that modulate sinking rates. Their increased process length relative to vesicle diameter could plausibly represent a hydrodynamic adaptation to staying suspended in the water column, which in turn could reflect an increasing adaptation to a planktonic lifestyle. It has been shown experimentally that cell morphotypes with numerous long spines promote suspension, although only if symmetrically arranged [110] and numerous long spines in modern foraminifera produce sufficient drag to impede settling through the water column [111]. Similarly, spines on the eggs of planktic animals decrease sinking rates; immediately hatching eggs are often smooth walled, while over-wintering eggs possess spines to provide a delayed sinking mechanism and facilitate embryonic development [107,112]. Conversely, resting stages of meroplankton—organisms that inhabit both the surface waters and benthos at different life stages—are often ornamented such that sinking rates are increased. Short, calcified spines of certain dinoflagellate resting cysts help to increase settling velocity [113] and processes on diatoms (in conjunction with extracellular polymeric substances) can serve to increase sinking rates through the promotion of entanglement and aggregation [112,114], although diatoms with sufficiently long processes show slower settling velocities [115]. Recent work in computational fluid dynamics highlights the potential of this method for better understanding the ecology of ancient organisms [116–119] and could potentially shed light on the adaptation of new forms by late Ediacaran and Cambrian protists.

An alternative explanation for changes in organic-walled microfossil morphology centres on predation. It has been suggested that early Cambrian acanthomorphic organic-walled microfossils evolved spines in direct response to grazing by primary consumers [23], reflected in the contemporaneous small carbonaceous fossil (SCF) record of the remains of early animals (e.g. [120]). However, we argue that this explanation has weaknesses when viewing morphological trends across longer temporal scales. All 14 of the process morphologies seen in early Cambrian organic-walled microfossils first appear in the Mesoproterozoic or Neoproterozoic [33], implying that, if spines evolved as a mechanism for deterring grazing, organic-walled microfossils were already subject to predation before the rise of metazoans. In fact, evidence for predation can be found in organic-walled microfossils from multiple assemblages, suggesting that eukaryotes were actively preyed upon by other eukaryotes [41,121,122]. Instead, as hypothesized by Butterfield [23] and Wallet *et al.* [123], spines (in conjunction with extracellular polymeric substances) may have evolved to evade predation *indirectly*; not by preventing ingestion but through increasing the chances of aggregation and therefore sinking rates to refugia in deeper waters, which would allow meroplankton to complete their life cycles and ensure the seeding of future generations. Moreover, both the reduction of cell size, and/or increase of effective cell size (through proportionately longer processes) may also be another *indirect* evasion of predation, as planktonic predators adhere to specific size ratios when selecting their prey [124].

## 5. Conclusion

In summary, our data provide evidence for a pronounced and sustained shift in size and morphological disparity among organic-walled microfossils beginning in the late Ediacaran and continuing into the Cambrian. Specifically, we show that vesicle diameter decreases, the ratio of process length to vesicle diameter increases, and occupied morphological space shifts from large morphotypes with short and diverse process shapes to small morphotypes with relatively long, thin processes with diverse tip shapes, as well as flange and colonial/aggregate morphotypes. While a significant size change in organic-walled microfossils across the Precambrian–Cambrian boundary has been previously documented, our data demonstrate that this shift begins in the Ediacaran following the Shuram excursion, and is accompanied by an increase in relative process length after the Ediacaran-Cambrian transition, which has not been previously quantified. Crucially, our study suggests broad temporal correlation—and plausible mechanistic links—between environmental perturbations and morphological change in Neoproterozoic/early Cambrian microfossils. The morphological shift illustrated here—which may have been influenced by nutrient limitation—would likely also have had an important impact on hydrodynamics, perhaps reflecting a shift to more planktonic morphotypes and lifestyles. Planktonic microbial eukaryotes are foundational to modern marine food webs and play an essential role in governing biogeochemical cycles and carbon export; our results highlight the expansion of eukaryotes into the plankton and their emerging role in the evolution of modern marine ecology at the Ediacaran–Cambrian transition.

## Data Availability

Data for this study are available in the Dryad repository [125]. This repository contains the full Ediacaran-Cambrian microfossil and median ages datasets and the R scripts needed to produce each analysis and produce the main text and supplemental figures. Supplementary material is available online [126].
